# Exercise-Associated Syncope: A Narrative Review of Pathophysiology, Clinical Classification, and the Diagnostic Role of the Head-Up Tilt Test

**DOI:** 10.7759/cureus.113720

**Published:** 2026-07-31

**Authors:** Sebastian Asteguieta, Cesar Lopez, Carlos Diaz

**Affiliations:** 1 Research, Universidad Francisco Marroquín, Guatemala City, GTM; 2 Cardiology, Cardio Care, Guatemala City, GTM; 3 Faculty of Medicine, Universidad Francisco Marroquín, Guatemala City, GTM

**Keywords:** autonomic dysfunction, exercise syncope, head-up tilt test, syncope, vasovagal syncope

## Abstract

Syncope is a common clinical condition characterized by a transient loss of consciousness caused by temporary global cerebral hypoperfusion. Exercise-associated syncope is clinically important because it may reflect benign physiological mechanisms or represent the initial manifestation of a potentially life-threatening cardiovascular disorder. This narrative review examines current evidence on its epidemiology, pathophysiology, classification, diagnostic evaluation, differential diagnosis, and management. The reviewed evidence indicates that syncope occurring during exercise should be considered a high-risk presentation requiring prompt cardiovascular evaluation, whereas post-exercise episodes are more frequently associated with reflex mechanisms and impaired venous return. Initial assessment should include a detailed history, physical examination, orthostatic blood pressure measurement, and 12-lead electrocardiography, with echocardiography, exercise testing, ambulatory rhythm monitoring, or head-up tilt testing selected according to the suspected mechanism. Head-up tilt testing is most useful when reflex syncope remains suspected after the initial evaluation. A structured, risk-based diagnostic approach is therefore recommended to identify cardiac causes early while avoiding unnecessary testing in patients with likely benign syncope.

## Introduction and background

Syncope is defined as a transient loss of consciousness caused by global cerebral hypoperfusion, characterized by rapid onset, short duration, and spontaneous recovery [1]. It represents a common clinical problem, accounting for approximately 1-3% of emergency department visits and up to 6% of hospital admissions, according to contemporary epidemiological analyses and systematic reviews [1,2]. Meta-analyses evaluating population-based cohorts estimate that nearly one-third of individuals will experience at least one syncopal episode during their lifetime, highlighting the substantial clinical and public health relevance of this condition [2].

Recent evidence from systematic reviews and meta-analyses has improved the understanding of the underlying mechanisms of syncope. Current literature indicates that the pathophysiology of syncope is primarily related to a transient reduction in cerebral blood flow secondary to systemic hypotension or a sudden decrease in cardiac output [3]. Physiological studies demonstrate that cerebral autoregulation maintains relatively stable cerebral perfusion across a range of arterial pressures; however, abrupt hemodynamic changes may exceed these compensatory mechanisms and lead to transient cerebral hypoperfusion and loss of consciousness [3,4].

Among the different clinical presentations, exercise-associated syncope has received particular attention in recent years because of its potential association with serious cardiovascular disease. Evidence derived from contemporary cohort studies and systematic reviews suggests that syncope occurring during physical exertion may be related to structural heart diseases such as hypertrophic cardiomyopathy, severe aortic stenosis, or arrhythmogenic cardiomyopathy, as well as malignant arrhythmias that significantly increase the risk of sudden cardiac death [5,6]. Consequently, current guideline-based reviews emphasize that syncope during exercise should be considered a high-risk clinical presentation until cardiac etiologies have been excluded [6].

In contrast, syncope occurring immediately after exercise is more frequently associated with benign autonomic mechanisms. Recent systematic reviews describe post-exercise syncope as primarily related to persistent peripheral vasodilation and reduced venous return following abrupt cessation of muscular activity, which can result in reflex-mediated hypotension and cerebral hypoperfusion [4,7]. Differentiating these mechanisms from potentially life-threatening cardiac causes remains a critical aspect of clinical evaluation.

Advances in diagnostic strategies have also been reported in recent literature. Evidence from systematic reviews and clinical studies indicates that the head-up tilt test remains one of the most valuable non-invasive tools for evaluating patients with suspected reflex syncope and autonomic dysfunction [8-11]. The test reproduces orthostatic stress under controlled conditions, allowing clinicians to observe hemodynamic responses and classify vasovagal syncope into vasodepressor, cardioinhibitory, or mixed patterns based on blood pressure and heart rate changes [8]. Furthermore, recent analyses have suggested that combining tilt testing with additional physiological monitoring techniques may improve diagnostic accuracy in patients with unexplained syncope [9,11-14].

Despite these advances, the available literature often evaluates exertional syncope, post-exercise syncope, and the diagnostic role of the head-up tilt test separately. Consequently, an integrated clinical framework that clearly distinguishes these presentations and defines the appropriate role of tilt testing within their diagnostic evaluation remains insufficiently developed. This narrative review aims to critically summarize the current evidence regarding the epidemiology, pathophysiology, clinical classification, differential diagnosis, and evaluation of exercise-associated syncope, with particular emphasis on distinguishing syncope occurring during exercise from that occurring immediately after exercise and clarifying the diagnostic role and limitations of the head-up tilt test. By integrating cardiovascular, autonomic, and exercise-related mechanisms, this review seeks to provide clinicians with a practical framework for risk stratification and diagnostic decision-making.

## Review

Materials and methods

This narrative review was conducted through a structured search of the biomedical literature to identify relevant publications on exercise-associated syncope and the diagnostic role of the head-up tilt test. The literature search was performed between March 10 and March 20, 2025, using PubMed/Medical Literature Analysis and Retrieval System Online (MEDLINE), the Cochrane Library, and Google Scholar.

The search strategy combined Medical Subject Headings (MeSH) and free-text terms, including “syncope,” “exercise-associated syncope,” “exercise syncope,” “exertional syncope,” “vasovagal syncope,” “reflex syncope,” “orthostatic hypotension,” “cardiac syncope,” and “head-up tilt test,” using the Boolean operators AND and OR.

Articles published in English between January 2022 and March 2025 were considered. Eligible publications included clinical practice guidelines, systematic reviews, meta-analyses, randomized controlled trials, observational cohort studies, and narrative reviews addressing the epidemiology, pathophysiology, classification, diagnosis, or management of exercise-associated syncope. Seminal clinical practice guidelines and consensus statements published before 2022 were also considered when required to describe established standards of care or provide essential historical context. Editorials, conference abstracts, duplicate publications, case reports, and studies unrelated to exercise-associated syncope were excluded.

Title and abstract screening, full-text assessment, and final study selection were performed by one reviewer. No reviewer disagreements occurred. Consequently, no formal process for resolving disagreements between reviewers was applicable. The final selection was subsequently discussed with the coauthors to confirm its relevance to the objectives of the review. Publications were selected according to prespecified eligibility criteria, relevance to exercise-associated syncope, study design, and clinical applicability. Because this was a narrative rather than a systematic review, no formal risk-of-bias or methodological quality-appraisal instrument, such as A MeaSurement Tool to Assess Systematic Reviews 2 (AMSTAR-2), was applied. Therefore, the included publications were not assigned quality ratings, and the findings are presented as a qualitative narrative synthesis rather than as a graded assessment of evidence certainty. A total of 18 publications were included in the final narrative synthesis.

UpToDate was consulted separately after study selection solely to contextualize established clinical recommendations and verify consistency with contemporary clinical practice. It was not searched as an evidence database, did not contribute publications to the final study sample, and was not used as a substitute for the primary studies, systematic reviews, or clinical guidelines included in the narrative synthesis. Because this was a narrative review, no formal risk-of-bias or methodological quality-appraisal tool was applied. 

This study was conducted as a narrative review and was not designed as a systematic review; therefore, formal adherence to Preferred Reporting Items for Systematic Reviews and Meta-Analyses (PRISMA) guidelines was not required. A literature search was performed using PubMed and the Cochrane Library for English-language publications addressing exercise-associated syncope, exertional syncope, post-exertional syncope, head-up tilt testing, autonomic dysfunction, and cardiac causes of syncope. UpToDate was consulted only as a tertiary resource to identify relevant concepts and primary references. Titles and abstracts were screened by one reviewer, and articles were selected according to their clinical relevance, methodological quality, and contribution to the objectives of the review. Priority was given to clinical guidelines, consensus statements, systematic reviews, and recent original studies. No formal risk-of-bias assessment was performed because of the narrative nature of the review.

Exercise-associated syncope

Physical exercise produces profound physiological changes in the cardiovascular and autonomic systems that are necessary to meet the increased metabolic demands of skeletal muscles. During dynamic exercise, oxygen consumption increases significantly, requiring a coordinated cardiovascular response to maintain adequate tissue perfusion and systemic arterial pressure. One of the most important cardiovascular adaptations is the increase in cardiac output, which may rise approximately four- to six-fold during maximal exercise in healthy individuals. This response results from combined increases in heart rate and stroke volume [1]. This increase occurs through a combination of elevated heart rate and enhanced stroke volume mediated by sympathetic nervous system activation [2,4].

At the same time, exercise induces peripheral vasodilation within active skeletal muscles, which facilitates oxygen delivery and metabolic exchange. Local metabolic factors, including increased carbon dioxide concentration, decreased oxygen tension, and accumulation of metabolites such as lactate and adenosine, contribute to the relaxation of vascular smooth muscle within exercising muscle groups. Although this vasodilation decreases systemic vascular resistance, arterial pressure is typically maintained through a compensatory increase in cardiac output and sympathetic-mediated vasoconstriction in non-exercising vascular beds [4,5].

Another physiological mechanism involved in exercise is the skeletal muscle pump. Rhythmic contractions of the lower-extremity muscles compress the veins and, together with competent venous valves, facilitate the movement of blood toward the heart. This mechanism contributes to venous return during dynamic exercise, although its relative contribution may vary according to exercise intensity, body position, and the muscle groups involved [8].

Autonomic regulation also plays a crucial role in the cardiovascular response to exercise. Sympathetic nervous system activation leads to increased heart rate, myocardial contractility, and peripheral vasoconstriction in certain vascular territories, while parasympathetic tone is simultaneously withdrawn. These adjustments ensure that systemic arterial pressure remains stable despite the substantial redistribution of blood flow toward active muscles [5]. Baroreceptor reflexes located in the carotid sinus and aortic arch continue to modulate these responses, allowing rapid adaptation to changing hemodynamic conditions during physical exertion.

Immediately after exercise, cessation of the skeletal muscle pump reduces the mechanical support for venous return, while vasodilation in previously active muscles may persist. This combination promotes peripheral venous pooling, reduces cardiac preload and stroke volume, and may transiently compromise arterial pressure and cerebral perfusion [6,7]. Dehydration, heat exposure, and abrupt termination of exercise may further accentuate these hemodynamic changes. Consequently, post-exercise syncope is more commonly associated with reflex mechanisms and transient hemodynamic instability. In contrast, syncope occurring during exercise is considered a higher-risk presentation because it may indicate structural heart disease, outflow obstruction, myocardial ischemia, or a malignant arrhythmia and therefore warrants prompt cardiovascular evaluation [8,9].

Major syncope guidelines identify syncope occurring during exertion as a high-risk feature that warrants targeted investigation for structural heart disease and clinically significant arrhythmias. Initial assessment should include a detailed clinical history, physical examination, orthostatic blood pressure measurement, and 12-lead electrocardiography, followed by echocardiography, exercise testing, ambulatory rhythm monitoring, or advanced cardiac imaging according to the suspected mechanism [3,8].

Thermoregulatory mechanisms during exercise may further influence cardiovascular responses. Increased body temperature promotes additional peripheral vasodilation, particularly in cutaneous circulation, to facilitate heat dissipation. While this response is essential for maintaining thermal balance, it may contribute to reductions in systemic vascular resistance and circulating central blood volume, especially during prolonged or high-intensity exercise [8]. Dehydration and fluid loss through sweating may further exacerbate these effects by reducing intravascular volume.

The major causes of exercise-associated syncope differ according to the timing of the event, associated clinical manifestations, underlying mechanisms, and level of cardiovascular risk. A structured comparison of these conditions is provided in Table 1 to facilitate their clinical differentiation.

**Table 1:** Comparison of the major causes of exercise-associated syncope Major clinical characteristics and diagnostic approaches for common causes of exercise-associated syncope. Syncope occurring during exercise should raise greater concern for cardiac disease, whereas episodes immediately after exercise are more commonly associated with reflex mechanisms or post-exercise hypotension. BP: blood pressure; ECG: electrocardiogram; POTS: postural orthostatic tachycardia syndrome

Cause	Typical timing	Clinical clues	Principal evaluation	Risk level
Reflex/vasovagal syncope	Usually, immediately after exercise	Prodrome, nausea, warmth, diaphoresis, prolonged standing	History, ECG, orthostatic measurements; tilt testing in selected cases	Usually low
Post-exercise hypotension	Immediately after abrupt exercise cessation	Venous pooling, dehydration, rapid recovery when supine	History, blood pressure assessment, and active standing	Usually low
Orthostatic hypotension	After standing or during recovery	Lightheadedness, medication use, and autonomic symptoms	Supine and standing BP; autonomic testing when indicated	Variable
POTS/orthostatic intolerance	During standing or after exercise	Palpitations, fatigue, exercise intolerance without marked hypotension	Active standing or tilt-table testing	Usually low, but symptomatic
Structural cardiac disease	During exertion	Murmur, chest pain, dyspnea, abnormal ECG, or family history	ECG, echocardiography, exercise testing, and cardiac imaging	High
Cardiac arrhythmia	During exertion, often sudden	Minimal prodrome, palpitations, family history of sudden death	ECG, exercise testing, and ambulatory monitoring	High
Inherited arrhythmogenic syndrome	During exercise or emotional stress	Young age, exertional palpitations, family history, abnormal or sometimes normal resting ECG	Exercise testing, ambulatory monitoring, and targeted genetic evaluation	High
Neurological or psychogenic event	Variable	Prolonged episode, atypical movements, or inconsistent hemodynamic changes	Neurological assessment; video-EEG or tilt testing in selected cases	Variable

As summarized in Table 1, syncope occurring during exercise, particularly when sudden and unaccompanied by a typical reflex prodrome, should raise concern for structural heart disease, cardiac arrhythmias, or inherited arrhythmogenic syndromes. In contrast, post-exertional syncope is more commonly associated with reflex mechanisms, persistent peripheral vasodilation, venous pooling, dehydration, or orthostatic intolerance. Nevertheless, the timing of the event should be interpreted together with the patient’s clinical history, physical examination, electrocardiographic findings, and family history.

**Figure 1:**
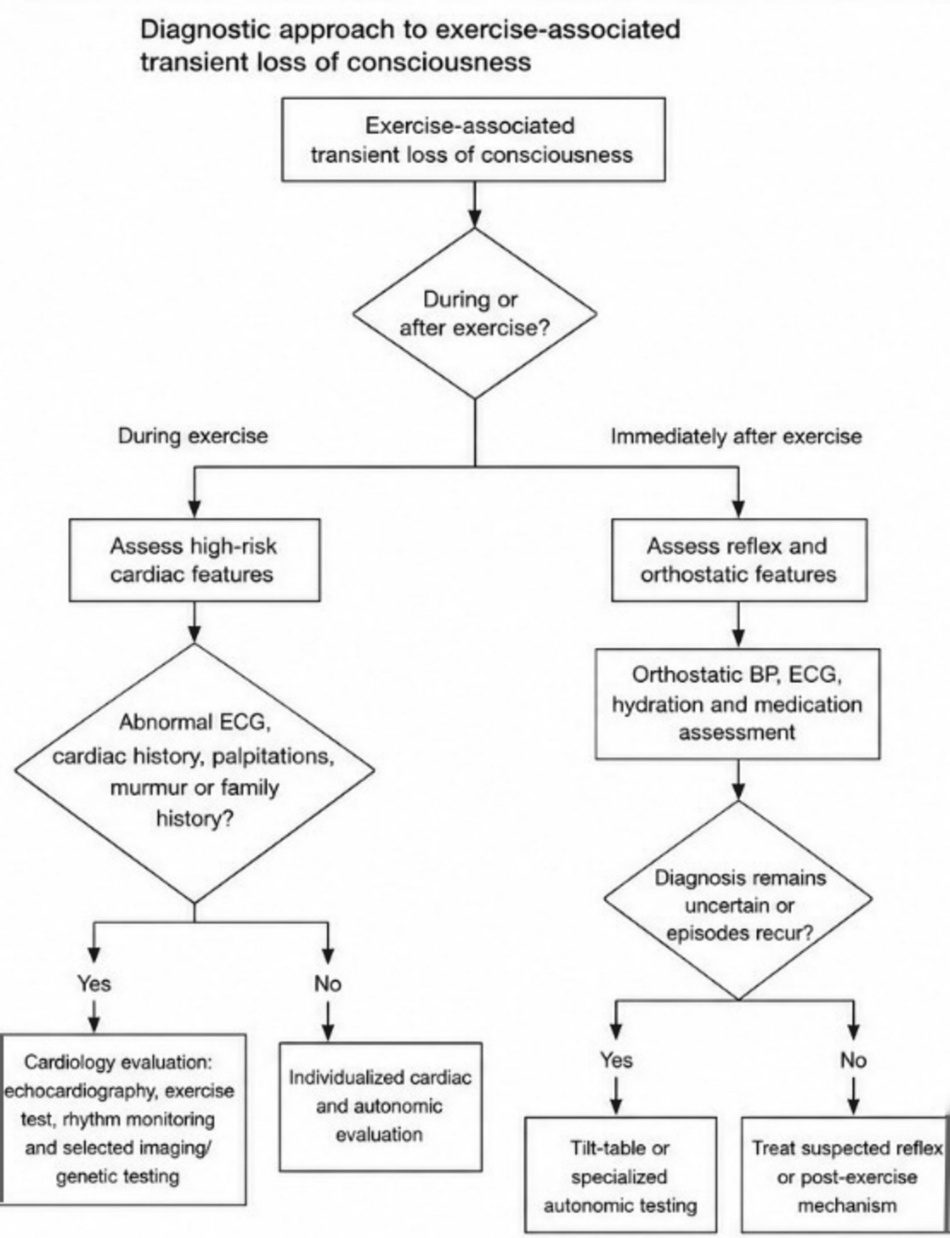
Diagnostic algorithm for the evaluation of exercise-associated syncope Proposed diagnostic approach to exercise-associated syncope. Syncope during exertion, particularly without a typical reflex prodrome, warrants investigation for structural or arrhythmic cardiac disease. Post-exertional episodes are more commonly associated with reflex mechanisms, venous pooling, dehydration, or orthostatic intolerance. The selection of advanced cardiac, autonomic, neurological, or genetic testing should be guided by the clinical phenotype. The figure was created using Canva (Canva Pty Ltd., Sydney, New South Wales, Australia).

Given the distinct risk profiles of syncope occurring during versus immediately after exercise, a structured diagnostic approach incorporating event timing and cardiovascular warning signs is proposed in Figure 1.

Classification of syncope

From a clinical perspective, syncope can be categorized according to the predominant cardiovascular mechanism responsible for the transient reduction in cerebral perfusion. Contemporary clinical frameworks generally divide syncope into reflex syncope, orthostatic hypotension-related syncope, and cardiac syncope. This classification allows clinicians to organize diagnostic evaluation and differentiate conditions that are generally benign from those that may be associated with increased cardiovascular risk [2,7].

Reflex syncope encompasses a group of conditions in which transient autonomic dysregulation produces abrupt cardiovascular changes leading to loss of consciousness. The most common entity within this group is neurocardiogenic syncope, also commonly referred to as vasovagal syncope. In this condition, autonomic reflex pathways produce a sudden shift toward parasympathetic dominance accompanied by withdrawal of sympathetic activity. This imbalance alters cardiovascular stability and may lead to rapid circulatory changes that compromise cerebral perfusion [5,7]. Neurocardiogenic syncope frequently manifests in individuals without structural heart disease and represents a large proportion of syncopal events in younger populations.

Hemodynamic responses observed during diagnostic evaluation allow further characterization of neurocardiogenic syncope into specific physiological patterns. One recognized subtype is cardioinhibitory syncope, in which excessive vagal activation produces pronounced slowing of the sinus node or transient impairment of atrioventricular conduction. In such situations, the resulting bradycardia may significantly reduce effective cardiac output. Another form is vasodepressor syncope, which is primarily driven by peripheral vasodilation and reduction of systemic vascular resistance, while heart rate remains relatively preserved. A third pattern, referred to as mixed syncope, involves simultaneous contributions from both mechanisms, with reductions in arterial pressure occurring together with varying degrees of bradycardia [7,9].

Additional entities included within reflex syncope are characterized by specific triggering circumstances that activate autonomic reflexes affecting cardiovascular regulation. In situational syncope, particular physiological activities can provoke transient autonomic responses that influence vascular tone or cardiac rhythm. Carotid sinus syncope, another reflex-mediated condition, arises from exaggerated sensitivity of the baroreceptors located in the carotid sinus region. Mechanical stimulation in susceptible individuals can produce abrupt reflex cardiovascular inhibition and transient circulatory instability [5,7].

A second major category involves syncope associated with orthostatic circulatory disturbances. In these cases, inadequate hemodynamic adaptation to upright posture results in insufficient maintenance of arterial pressure. Several clinical forms have been described, including classical orthostatic hypotension, delayed orthostatic hypotension, and syndromes characterized by abnormal autonomic regulation, such as postural orthostatic tachycardia syndrome. These conditions may occur in association with neurodegenerative disorders affecting autonomic pathways, metabolic diseases, aging-related autonomic impairment, or pharmacologic effects that interfere with vascular tone regulation [8,9].

The third category includes cardiac syncope, in which transient circulatory failure arises from abnormalities affecting cardiac rhythm or structural integrity of the heart. In contrast to reflex forms, the mechanism here involves an inability of the heart to maintain effective forward blood flow due to disturbances in electrical conduction or mechanical obstruction to circulation. Because these conditions may reflect significant cardiovascular disease, their identification carries important implications for prognosis and clinical management [6,7].

Inherited arrhythmogenic syndromes should also be considered in patients with exertional syncope, particularly when episodes occur without a typical reflex prodrome or are accompanied by exertional palpitations or a family history of premature sudden cardiac death. Exercise may precipitate ventricular arrhythmias in conditions such as catecholaminergic polymorphic ventricular tachycardia and certain long-QT syndrome phenotypes. Appropriate evaluation may include exercise testing, ambulatory electrocardiographic monitoring, cardiac imaging, and targeted genetic assessment when supported by the clinical phenotype and family history [2,3].

Role of the head-up tilt test

The head-up tilt test has been used in the evaluation of unexplained syncope since its early clinical application demonstrated that controlled orthostatic stress could reproduce vasovagal episodes in susceptible patients. Its contemporary role is primarily focused on patients with suspected reflex syncope when the diagnosis remains uncertain after the initial clinical evaluation. The objective is not merely to provoke syncope but to reproduce recognizable symptoms while documenting the associated blood pressure and heart rate responses [9,10].

Contemporary autonomic assessment extends beyond conventional tilt-table measurements of intermittent blood pressure and heart rate. Depending on the clinical presentation and available expertise, tilt testing may be combined with continuous beat-to-beat blood pressure monitoring, electrocardiography, active standing, autonomic function tests, video recording, electroencephalography, end-tidal carbon dioxide measurement, or selected cerebral perfusion measurements. These additions may improve the characterization of reflex syncope, orthostatic hypotension, postural orthostatic tachycardia syndrome, and psychogenic transient loss of consciousness; however, their use should be individualized, and several remain primarily restricted to specialized autonomic laboratories [2,9].

The diagnostic performance of the head-up tilt test varies according to the protocol used. Pharmacological provocation with sublingual nitroglycerin may increase test sensitivity compared with passive tilting, although this improvement may be accompanied by some reduction in specificity. A meta-analysis of 55 studies reported that protocols incorporating nitroglycerin had a sensitivity of 66% (95% CI: 60%-72%) and the highest diagnostic odds ratio among the evaluated protocols. However, diagnostic estimates varied across studies according to patient characteristics, tilt angle, test duration, nitroglycerin dose, and the criteria used to define a positive response [9]. Therefore, numerical estimates should be interpreted in relation to the specific protocol and population studied.

Recent research has examined whether early physiological changes during tilting can predict the final test result. One observational study reported that, among female patients with syncope, a heart rate increase of approximately 12 beats per minute within the first 10 minutes after tilting was independently associated with a positive head-up tilt test. Because this threshold was derived from a specific study population, it should be interpreted as a preliminary predictive marker rather than a universally validated diagnostic cutoff [6].

Another area of recent investigation involves the role of baroreflex sensitivity in predicting tilt-induced syncope. Contemporary studies have reported higher baseline baroreflex sensitivity in patients with suspected vasovagal syncope than in control populations. Increased baroreflex responsiveness has been associated with a greater likelihood of positive tilt test responses and with the occurrence of cardioinhibitory events during testing, suggesting that abnormal reflex control of blood pressure may play a significant role in susceptibility to reflex syncope [5].

Another important role of tilt testing in contemporary clinical practice is its ability to assist in the differentiation of syncope from other causes of transient loss of consciousness. In patients with recurrent unexplained episodes, the test may help distinguish reflex syncope from conditions such as psychogenic pseudosyncope or certain forms of orthostatic intolerance. When interpreted alongside clinical history and other diagnostic findings, tilt testing provides valuable information that contributes to a more precise diagnostic assessment [15,16].

Emerging exercise-specific physiological and diagnostic considerations

Recent investigations have expanded the evaluation of exercise-associated syncope beyond conventional heart rate recovery and tilt-table testing. One emerging area is the analysis of RR-interval kinetics during the immediate post-exercise period. Exercise cessation produces rapid sympathetic withdrawal and progressive parasympathetic reactivation, resulting in a nonlinear increase in RR intervals during recovery. Traditional heart rate recovery measurements capture only selected time points, whereas analysis of RR-interval variability and complexity may provide additional information regarding dynamic cardiac autonomic regulation. In young adults, complexity measures obtained during the first five minutes after maximal exercise provided information that was not fully represented by conventional heart rate recovery indices [17]. Nevertheless, these methods remain primarily investigational, and their ability to identify patients susceptible to post-exercise reflex syncope has not been prospectively established.

The exercise pressor reflex represents another relevant mechanism. Mechanical and metabolic stimulation of group III and IV skeletal-muscle afferents activates the muscle mechanoreflex and metaboreflex, respectively. Together with central command and arterial baroreflex resetting, these pathways increase sympathetic outflow, heart rate, vascular resistance, and arterial pressure during exercise. This coordinated response helps maintain systemic pressure despite marked vasodilation in active skeletal muscle. Abnormal regulation of the exercise pressor reflex has been described in several cardiovascular and metabolic disorders, although its specific contribution to exercise-associated syncope remains incompletely defined [18]. From a mechanistic perspective, inadequate integration of central command, baroreflex responses, and muscle afferent signaling could impair cardiovascular compensation during exertion or early recovery. Further studies are needed before exercise pressor reflex measurements can be incorporated into routine syncope evaluation.

Dynamic cerebral autoregulation may also influence individual susceptibility to exercise-associated loss of consciousness. Cerebral perfusion during and after exercise depends on the interaction between arterial pressure, arterial carbon dioxide tension, cardiac output, cerebrovascular resistance, and the capacity of cerebral vessels to buffer rapid blood pressure fluctuations. Cerebral blood flow responses vary according to exercise intensity and modality, and substantial hemodynamic changes may occur during high-intensity aerobic or resistance exercise [19]. Following exercise cessation, systemic hypotension, persistent peripheral vasodilation, and changes in ventilation may transiently challenge cerebral perfusion. A systematic review demonstrated that several cerebral blood velocity and cerebrovascular reactivity measurements require variable periods to return to baseline after aerobic exercise, highlighting the dynamic nature of post-exercise cerebrovascular recovery [20]. However, transcranial Doppler and formal assessment of dynamic cerebral autoregulation remain research-oriented tools rather than standard tests for exercise-associated syncope.

A further diagnostic challenge is differentiating physiological cardiac adaptation to sustained athletic training from underlying cardiovascular pathology. Athletes may exhibit increased ventricular chamber dimensions, myocardial wall thickness, resting bradycardia, and electrocardiographic repolarization patterns that overlap with cardiomyopathy or inherited arrhythmogenic disease [21]. These findings should therefore be interpreted according to the athlete’s age, sex, ethnicity, sporting discipline, training exposure, symptoms, and family history. Exertional syncope, particularly when sudden or unaccompanied by a typical reflex prodrome, should not be attributed to athletic conditioning without an appropriate cardiovascular assessment. When initial electrocardiography and echocardiography are inconclusive, exercise testing, ambulatory rhythm monitoring, cardiac magnetic resonance imaging, or genetic evaluation may be considered according to the suspected disorder. Multimodality assessment is particularly important when physiological ventricular remodeling overlaps with features of dilated or arrhythmogenic cardiomyopathy [21,22]. These emerging physiological and diagnostic approaches complement rather than replace structured clinical assessment and may help distinguish benign post-exercise autonomic responses from potentially serious exertional cardiovascular disease.

Discussion

Syncope remains a common clinical problem with a broad differential diagnosis, making accurate identification of the underlying etiology essential for appropriate risk stratification and management. In the setting of exercise, the principal clinical challenge lies in distinguishing benign physiological or reflex-mediated mechanisms from potentially life-threatening cardiovascular disorders. Although exercise-associated syncope in otherwise healthy individuals is frequently related to neurally mediated reflexes or post-exercise hemodynamic changes, syncope occurring during physical exertion warrants a comprehensive cardiovascular evaluation because it may represent structural heart disease, inherited arrhythmogenic syndromes, or malignant cardiac arrhythmias associated with an increased risk of sudden cardiac death.

**Figure 2:**
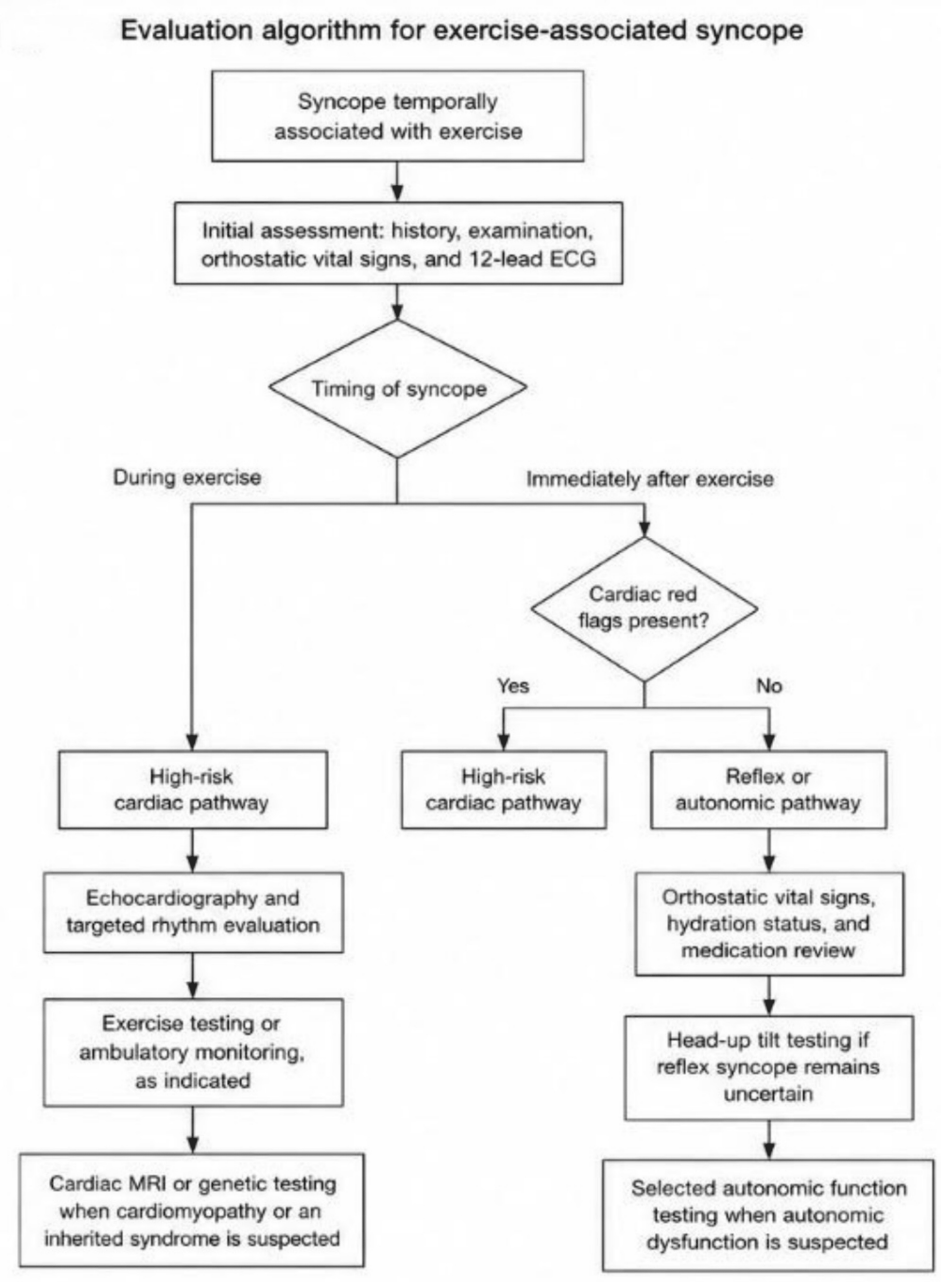
Proposed diagnostic approach to syncope temporally associated with exercise All patients should undergo an initial assessment comprising a detailed clinical history, physical examination, orthostatic vital signs, and 12-lead electrocardiography. Syncope occurring during exercise should be considered a high-risk presentation and should prompt targeted evaluation for structural heart disease and malignant arrhythmias. Syncope occurring immediately after exercise is more commonly associated with reflex or hemodynamic mechanisms; however, the presence of cardiac red flags should redirect the patient to the cardiac evaluation pathway. Cardiac magnetic resonance imaging and genetic testing should be reserved for patients with clinical, electrocardiographic, or familial findings suggestive of cardiomyopathy or an inherited arrhythmogenic syndrome. The figure was created using Canva (Canva Pty Ltd., Sydney, New South Wales, Australia). *Cardiac red flags: abnormal ECG, known structural heart disease, exertional chest discomfort or palpitations, syncope without prodrome, family history of premature sudden cardiac death, or suspected inherited cardiovascular disease.

A detailed clinical history remains the cornerstone of the diagnostic evaluation and should precede the use of complementary investigations. Information regarding the timing of the syncopal episode relative to exercise, precipitating factors, prodromal symptoms, duration of loss of consciousness, recovery characteristics, medication use, and family history of inherited cardiovascular disease or sudden cardiac death can substantially narrow the differential diagnosis and guide subsequent diagnostic testing. The principal clinical features, risk implications, and suggested diagnostic evaluation of exercise-associated syncope are summarized in Table 2. A practical diagnostic pathway based on the timing of syncope relative to exercise is presented in Figure 2. Syncope occurring during exertion should prompt a cardiac evaluation, whereas episodes occurring immediately after exercise are more commonly investigated through a reflex or autonomic pathway, provided that high-risk cardiovascular features are absent.

**Table 2:** Clinical differentiation and diagnostic evaluation of exercise-associated syncope Summary of the principal clinical presentations, risk implications, and targeted diagnostic evaluation of exercise-associated syncope. Syncope occurring during exercise should be treated as a high-risk presentation until structural and arrhythmic cardiac causes have been adequately excluded. In contrast, post-exercise syncope is more commonly associated with reflex and hemodynamic mechanisms.

Clinical presentation	Probable mechanism or causes	Suggestive clinical features	Risk implication	Suggested evaluation
Post-exercise reflex syncope	Persistent peripheral vasodilation, cessation of the skeletal muscle pump, venous pooling, and reduced preload	Occurs immediately after exercise cessation; prodromal nausea, warmth, diaphoresis, or lightheadedness; rapid and complete recovery	Usually lower risk when history and initial cardiovascular assessment are reassuring	Clinical history, physical examination, orthostatic blood pressure measurement, and 12-lead ECG; head-up tilt testing when reflex syncope remains suspected but uncertain
Syncope during exercise	Structural heart disease, outflow obstruction, myocardial ischemia, or malignant arrhythmia	Occurs during active exertion; may be sudden and without prodrome; possible chest discomfort, palpitations, dyspnea, or family history of sudden cardiac death	High-risk presentation requiring prompt cardiovascular evaluation	12-lead ECG and echocardiography, followed when indicated by exercise testing, ambulatory rhythm monitoring, cardiac imaging, or specialist electrophysiological evaluation
Orthostatic syncope associated with exercise	Volume depletion, dehydration, autonomic dysfunction, or medication-related hypotension	Symptoms after standing, prolonged exertion, heat exposure, or inadequate fluid intake; orthostatic blood pressure reduction	Risk depends on the underlying cause and associated comorbidities	Orthostatic blood pressure measurement, medication review, assessment of hydration status, and autonomic testing when clinically indicated
Reflex syncope unrelated to exertional cardiac disease	Vasovagal or situational autonomic reflex	Recognizable trigger, progressive prodrome, absence of structural heart disease, and complete recovery	Generally favorable prognosis after cardiac causes have been excluded	History-based assessment; head-up tilt testing when the diagnosis remains uncertain, or reproduction of symptoms is clinically useful
Non-syncopal transient loss of consciousness	Epileptic seizure, psychogenic pseudosyncope, metabolic disturbance, or other neurological cause	Prolonged loss of consciousness, atypical motor activity, delayed recovery, inconsistent hemodynamic findings, or absence of documented hypotension	Variable; requires evaluation according to the suspected alternative diagnosis	Witness history, neurological or metabolic assessment, and tilt testing with additional monitoring in selected cases

The literature included in this narrative review suggests the clinical value of a structured diagnostic approach that integrates comprehensive clinical assessment with targeted complementary investigations based on the suspected underlying mechanism [23]. Within this framework, the head-up tilt test remains an important diagnostic tool in patients with suspected reflex syncope when the diagnosis is uncertain after the initial evaluation [23-26]. In addition to reproducing symptoms under standardized conditions, tilt table testing provides valuable information regarding autonomic cardiovascular regulation and individual hemodynamic responses, thereby facilitating a more accurate understanding of the underlying pathophysiological mechanism [23,25].

When interpreted alongside the clinical history, physical examination, and electrocardiographic findings, the head-up tilt test can improve diagnostic confidence by confirming an autonomic mechanism and differentiating reflex syncope from other causes of transient loss of consciousness [23,26]. Furthermore, refinements in testing protocols, together with advances in the understanding of autonomic physiology, have enhanced its diagnostic performance and clinical utility, particularly in specialized syncope centers [23-26]. Nevertheless, the head-up tilt test should not be interpreted in isolation, and its findings should always be integrated with the overall clinical assessment to optimize diagnostic accuracy, risk stratification, and patient management [23-25].

Limitations

This narrative review has several limitations. Study screening, eligibility assessment, and article selection were performed by a single reviewer, which may have increased the risk of selection bias and reviewer subjectivity. In addition, no formal risk-of-bias or methodological quality-appraisal instruments were applied; consequently, the included publications were not assigned quality ratings, and the certainty of the evidence was not formally graded. Although multiple databases were searched using predefined eligibility criteria, the search was restricted by publication period and language, and relevant studies may therefore have been omitted. Despite the inclusion of selected recent literature on exercise-specific mechanisms, the review was not designed to provide an exhaustive synthesis of each emerging physiological domain, particularly emerging topics such as post-exercise heart rate recovery (HRR)-interval kinetics, the exercise pressor reflex, dynamic cerebral autoregulation, and the distinction between physiological athletic adaptation and cardiovascular pathology. Moreover, heterogeneity in study populations, exercise protocols, tilt-test methodologies, diagnostic definitions, and reported outcomes limited direct comparison across publications. UpToDate was consulted separately only to contextualize established clinical recommendations and was neither included in the study sample nor used as a substitute for primary evidence or clinical guidelines. Accordingly, the conclusions should be interpreted as a qualitative narrative synthesis of selected literature rather than as a comprehensive systematic review or a quantitatively graded assessment of the available evidence.

## Conclusions

In conclusion, exercise-associated syncope represents a clinically relevant presentation that requires careful evaluation to differentiate benign reflex mechanisms from potentially serious cardiovascular causes. Understanding the physiological effects of exercise on cardiovascular regulation and the mechanisms underlying different forms of syncope is essential for accurate diagnosis. Contemporary diagnostic strategies emphasize a comprehensive clinical assessment supported by targeted investigations, including head-up tilt testing with continuous beat-to-beat hemodynamic monitoring when reflex syncope is suspected. Emerging adjuncts, such as baroreflex sensitivity assessment, wearable photoplethysmography-based monitoring, and standardized autonomic symptom questionnaires, may further characterize autonomic dysfunction; however, their role in exercise-associated syncope requires additional validation, and they should not replace established clinical and cardiovascular evaluation. Continued research into autonomic regulation and cardiovascular responses to exercise may further improve the diagnosis and management of exercise-associated syncope. Future studies should evaluate the clinical utility of wearable monitoring technologies, advanced autonomic and continuous hemodynamic assessment, and precision risk-stratification approaches that integrate symptom timing, exercise characteristics, physiological measurements, cardiac imaging, and individual or familial risk factors. Prospective validation will be necessary to determine whether these strategies can improve early identification of high-risk cardiovascular conditions while avoiding unnecessary testing in patients with benign reflex or post-exercise syncope.
